# Photonics Scanning Pentaprism System for the Integrated Inspection of Large-Aperture Telescopes

**DOI:** 10.3390/s23156650

**Published:** 2023-07-25

**Authors:** Qichang An, Hanfu Zhang, Kun Wang, Xinyue Liu, Hongwen Li

**Affiliations:** 1 Changchun Institute of Optics, Fine Mechanics and Physics, Chinese Academy of Sciences, Changchun 130033, China; 2 Jilin Provincial Key Laboratory of Intelligent Wavefront Sensing and Control, Changchun 130033, China; 3 University of Chinese Academy of Sciences, Beijing 100039, China

**Keywords:** fiber-connection, spectrum calibration, assembling, integrated testing, large-aperture telescope

## Abstract

To improve their spatial resolution and detection capabilities, future ground-based optical telescopes will have a size of 30 m, and the aperture of space telescopes will be increased to 10 m. Such large optical systems necessitate the development of large integrated testing equipment. In this study, spectrum and system alignment measurements and wavefront quality checking were performed using the sub-aperture detection method and a fiber-connected Photonics Scanning Pentaprism (PSP). First, the system was aligned using an optical truss, ensuring that the optical axis was properly positioned. Second, using a sub-aperture light beam though the entrance pupil, light spots were formed on the focal plane and transmitted to the spectrometer via fibers to obtain the corresponding spectral components. Then, by taking measurements at different system positions, a full-aperture spectrum response could be reached. Lastly, by photon-integrated interference on the focal plane, intensity interference fringes could be projected at the entrance pupil of the system. And the wavefront quality of the system could be verified by observing the fringe deformation. The measurement accuracy of the optical axis of the system is better than 2 mrad. The spectral measurement accuracy was better than 5%, and the wavefront measurement accuracy surpassed 0.1 wavelengths (1 wavelength = 633 nm). This study effectively enhanced the detection and in situ calibration capabilities of large telescope systems, ensuring that the performance requirements can be met in the design of future telescopes.

## 1. Introduction

Future ground-based telescopes are expected to be 30–40 m in size. Meanwhile, the apertures of space telescopes are being increased from 6 to 10 m. In addition to providing a better understanding of the universe (including the “cosmic dark ages” and “first light”), large telescopes armed by adaptive optics facilitate significantly detailed observations of the structures of the universe. This is very significant for investigating black holes, dark matter, dark energy, exoplanets, and other important objects in astronomy. Telescopes must be tested during fabrication, as well as during the assessment of resembling. The following two operations are used for this testing: (1) the auto-collimation of planar mirrors and (2) planar wavefront emission by collimators. However, the costs and technical risks of building the corresponding large-aperture plane mirrors or collimators rapidly increase with the size of the aperture.

It is currently common to use sub-aperture stitching for such assessments. However, the use of small-aperture plane mirrors or collimators for stitching requires highly complex actuators [1]. To ensure the parallel accuracy of the optical axis during movement, a complex precision adjustment mechanism must be added, which increases the system error. 

The large optical test and integration site (LOTIS) system produced by Lockheed Martin is a representative example of the equipment used for in situ measurements. It has an aperture of 6.5 m and operating wavelength of 0.4–5 μm, and can achieve a root-mean-square (RMS) error of less than 85 nm for a wavefront, at a ±0.75 mrad field of view (FoV) [2]. To achieve the design accuracies and ensure the quality of the wavefront, the LOTIS system adopts 104 axial actuators behind the primary mirror to correct the surface shape and eliminate the influence of the primary mirror deformations on the wavefront. To ensure the quality of the wavefront, the system uses the following two pieces of in situ measurement equipment: a movable 1.8 m plane mirror for auto-collimation tests and a pentaprism for alignment tests [3].

Scanning pentaprisms are insensitive to tilt errors and have a good response to low-spatial-frequency signals. They are cost-effective and have good coverage and a simple structure. They are currently used for the testing of large planar and parabolic mirrors. The University of Arizona performed low-order tests of large-aperture plane mirrors with an accuracy of 18 nm. The LOTIS system wavefront cutting measurement equipment consists of nine pentaprism mirrors for optical pupil alignment, offering a measurement accuracy higher than 225 nm under atmospheric conditions. In addition to its other measurement equipment, LOTIS is equipped with visual and jitter measurement equipment for the mirror, which is used to ensure the normal operation of the measurement equipment [4].

Scanning pentaprisms can also be used to assess the parallelism of multi-axis optical systems. However, testing methods for the wavelength accuracy (i.e., 10% of the working wavelength) should be further explored. Wen Zhongkai et al. achieved a calibration accuracy of 0.998″ using bulk optical elements [5], Xu Danhui et al. used Newton’s rings interference fringes to obtain a parallelism measurement accuracy higher than 5″ [6], and Yi et al. designed an off-axis reflective large-aperture parallel light tube to achieve a multi-axis detection accuracy higher than 2.9226″ [7]. A spectral response test was employed to calibrate and correct the imaging performance by establishing the response relationship between a specific wavelength input at the pupil and the digital image output [8]. In addition, several performance evaluation parameters (e.g., the system response linearity, non-uniformity, signal-to-noise ratio, and dynamic range) could be obtained [9]. Currently, the following three technical approaches can be adopted: integrating sphere, collimator, and projection plane testing. Among these, the integrating sphere outputs the most uniform light [10]; however, it has a large volume and a limited opening diameter (an excessively large opening diameter affects the uniformity of the emergent light). Furthermore, integrating spheres with large diameters requires a combination of multiple light sources, and it is difficult to maintain the uniformity and consistency of the internal coatings of these spheres. In addition, the edge consistency of the integrating sphere outlet will be affected by its large diameter (its consistency with the Lambert radiator will trigger a significant deviation). The advantages of using a collimator for response testing include its controllable field of view and uniform energy distribution. However, in the future, optical equipment will have extremely large apertures exceeding 2 m (diameter) [11]. The cost and time required to manufacture collimators will be much higher, and they are difficult to move. The projection plane method can be adopted for the in situ testing of ground-based large-aperture survey telescopes, where the plane can be illuminated by light source projection. Furthermore, flat-field tests can be conducted inside a dome [12].

The traditional large-aperture telescope testing equipment uses volume optical elements. Different devices are used for different parameters, which are greatly affected by the environment, and it is difficult to meet the needs of in situ detection. Using photon scanning pentaprism can effectively reduce the volume and weight of the system. It can reduce the requirements of environmental conditions for testing and realize in-situ detection. By using the optical fiber interconnected system, the system stiffness can be used for reference transmission. It not only reduces the volume and weight of the measurement system, but also uses a common reference to transmit the metrology value, which reduces the accuracy loss caused by the non-common optical path difference and transmission link of the traditional method, effectively improves the in-situ measurement accuracy of the optical system, shortens the length of the traceability chain, and increases the efficiency and accuracy of the detection.

This study is organized using the following structure: Section 2 will introduce the measurement methods and principles, Section 3 will simulate and verify the principles of the system estimation technology, and Section 4 will conduct experimental verification of the optical axis, spectrum, and wavefront measurements.

## 2. Materials and Methods

### 2.1. Measurement Architecture and Methods

In this study, an improved Photonics Scanning Pentaprism (PSP) is proposed to perform waveguide-based photon collection and sensing, using the fiber-connected architecture. This addresses the significant effects of atmospheric and environmental vibrations on the optical path of conventional free-space scanning pentaprisms, which can make it impossible to test wavefront errors. Because the optical path of the PSP no longer had structural and spatial limitations, it was possible to arrange multiple short-range scanning mechanisms in a one-dimensional upward direction without introducing long rails. Moreover, the surface quality and stiffness requirements of the back-end mechanism could be reduced in response.

As a result, the testing and setup of large-aperture telescopic optical systems could be achieved at a low cost, reducing the dependence on large-aperture collimators. Furthermore, the separate small-aperture system was less affected by the atmosphere, which improved the testing accuracy. The evolution of the perceptual uncertainty of the parameters of the optical path delay and the influences of the errors on the final control performance must be clearly understood to achieve high-precision optical path control and error suppression in the frequency/spatial domain under multi-physics fields.

What is more, spectral measurements/calibration can also be performed and systems can be aligned using PSP. Stitching detection of the spectral response of the system can be achieved by taking measurements at different positions of the entrance pupil. By combining PSP with integrated interference, intensity interference fringes can be formed at the pupil. The light starts at the focal plane, passes through the system, and the fringe is obtained by a fiber-connected pentaprism system located at the pupil plane. Next, the wavefront slope is extracted. Finally, the wavefront information is reconstructed using the slope at that location.

**Figure 1:**
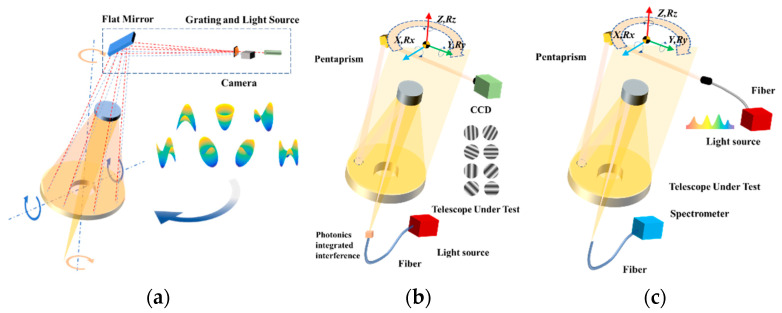
Architecture of Photonics Scanning Pentaprism (PSP) system: (**a**) establishment of telescope optical system baseline, (**b**) optical interference projection, and (**c**) spectral response measurement.

From Figure 1, it can be seen that the reference spatial coordinates of large-aperture optical elements can be established by using a multi-beam uniform Gaussian beam, as shown in Figure 1a. The local wavefront slope can be obtained by using stripe projection and camera acquisition, as shown in Figure 1b. The spectral response of the system can be obtained by utilizing the energy projection at the pupil and the backend spectrometer, as shown in Figure 1c.

### 2.2. Basic Principles of Wavefront Detection and Spectral Detection

Using the basic principles of Fourier optics, the complex optical field is analytically expressed through the light wave interference process for a single wavelength [13,14]. Therefore, a distribution model for the combined complex optical field in the non-narrowband case is obtained using the incoherent combination theory. The analytical expression of the combined light intensity and phase provides a theoretical analysis tool for subsequent studies. Specifically, using the complex optical field theory, the wavefront at a single wavelength, $W\left(u\right)$, can be expressed as follows:

(1) $$W\left(u\right)=E\exp\left[j\frac{2\pi A}{\lambda }\sin\left(2\pi uf\right)+\varphi \right]$$

where λ is the wavelength, A is the amplitude of the single-phase spatial frequency component, u indicates the intra-pupillary spatial coordinates, f denotes the spatial frequency domain coordinates, E denotes the aperture function, and φ denotes the initial phase.

The intensity can be obtained by squaring the modulus of the complex optical field as follows:

(2) $$I={\left|W\left(u\right)\right|}^{2}$$

After obtaining the expression for a single wavelength, the intensity of the interference between different wavelengths, $I\left(\lambda ,u\right)$, can be obtained as follows ($P\left(\lambda \right)$ is a matrix whose rows correspond to the spectral response function of the detector) [15,16,17]:

(3) $$I\left(\bar{\lambda },u\right)=\int_{-\infty }^{\infty }P\left(\lambda -\bar{\lambda }\right)I\left(\lambda ,u\right)d\lambda$$

where λ is the wavelength, and the filter function includes the shape of the passband and frequency response of the detector. Using these equations, we can obtain a quantitative description of the fringes arising from the two-path interference under non-narrow bands.

The wavefront slope at this location can be obtained through local interference fringes, and the system wavefront information can be obtained through the inverse solution of the fundamental principle of Fourier transform.

Here, $W\left(u\right)$ is found as follows [18,19]:

(4) $$W\left(u\right)=-\frac{j}{2\pi }F^{-1}\left\{\frac{F\left\{\frac{\partial W\left(u\right)}{\partial u}\right\}}{u}\right\}$$

## 3. Simulation Results

**Figure 2:**
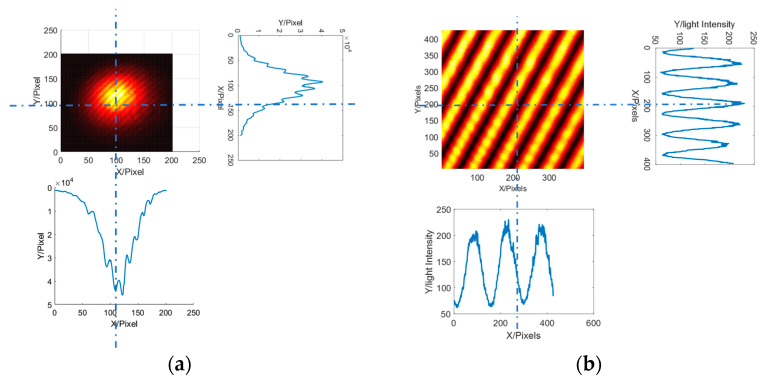
Two-dimensional single-frequency fringe: (**a**) interference of bulk optical elements, (**b**) integrated photon interference.

First, a two-dimensional interference fringe detection simulation model was constructed, where the characteristic frequency components in the two directions were obtained via a frequency domain analysis. The fringes obtained through the interference of volume optical elements and integrated photon interference are shown in Figure 2 and can be obtained through comparison. There is a significant uneven distribution of light intensity in the optical components. The uniformity and contrast of the fringes obtained by integrated photon interference are significantly higher than those obtained by bulk optical elements, and thus a higher-accuracy local wavefront slope measurement can be obtained.

**Figure 3:**
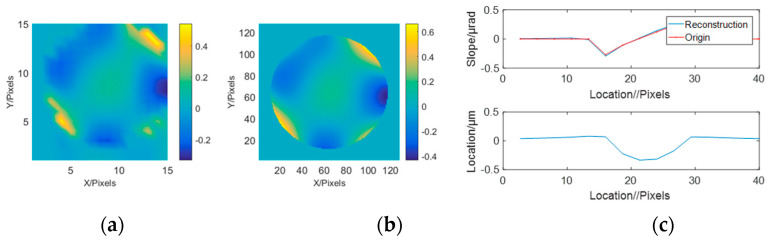

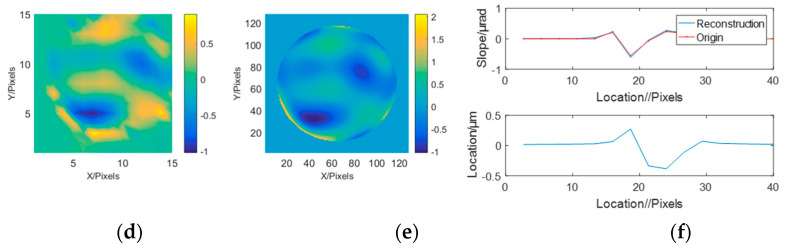
Slope reconstruction performances for the single mirror: (**a**) System wavefront restoration results for small aberration mode. (**b**) Raw wavefront in small aberration mode. (**c**) Section comparison in small aberration mode. (**d**) System wavefront restoration results for large aberration mode. (**e**) Raw wavefront in large aberration mode. (**f**) Comparison of sections in large aberration mode.

Reconstructed wavefront were computed based on slope sampling and reconstruction in the frequency domain. A slight degradation was observed only at the edges of the samples. The system wavefront restoration results for small aberration mode are shown in Figure 3a–c with a comparison of the original wavefront and section. The system wavefront restoration results for large aberration mode are shown in Figure 3d–f with a comparison of the original wavefront and section. The difference between the final reconstructed wavefront and the original broadcast is within 10%.

## 4. Experimental Verification

### 4.1. System Benchmark Establishment and Measurement

**Figure 4:**
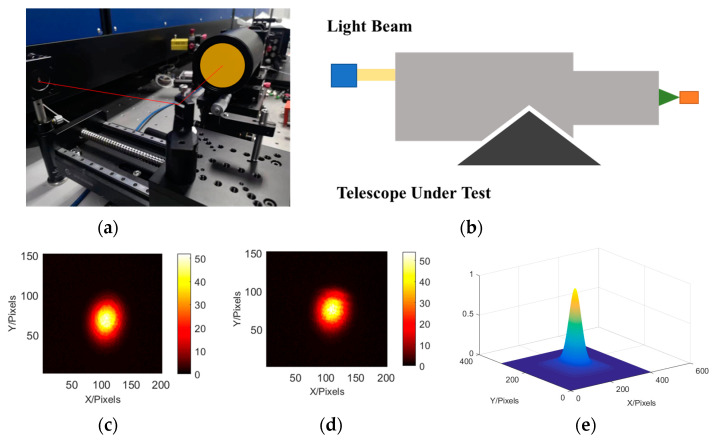
Optical axis detection: (**a**) system set-up, (**b**) device schematic, (**c**,**d**) system focal plane, (**e**) calculated light point position.

Here, the rotational symmetry of the system was used for testing. After obtaining the data in one position, the angle of the spectral grating was rotated to obtain circumferential sampling. By cross-comparison with data from the initial position, we ensured that the curvature radius and relative alignment of the optical element satisfied the requirements [20,21,22]. Various optical systems could be sequentially measured and their optical axes could be aligned using a mobile mechanism. The system’s optical axis parallelism measurement is shown in Figure 4. The scanning pentaprism can be used to obtain its light spot on the focal plane of the system, and the high-precision positioning of the system can be obtained using the correlation operation. The system’s optical axis positioning accuracy (2 mrad) can be obtained based on the pixel-to-focal ratio.

**Figure 5:**
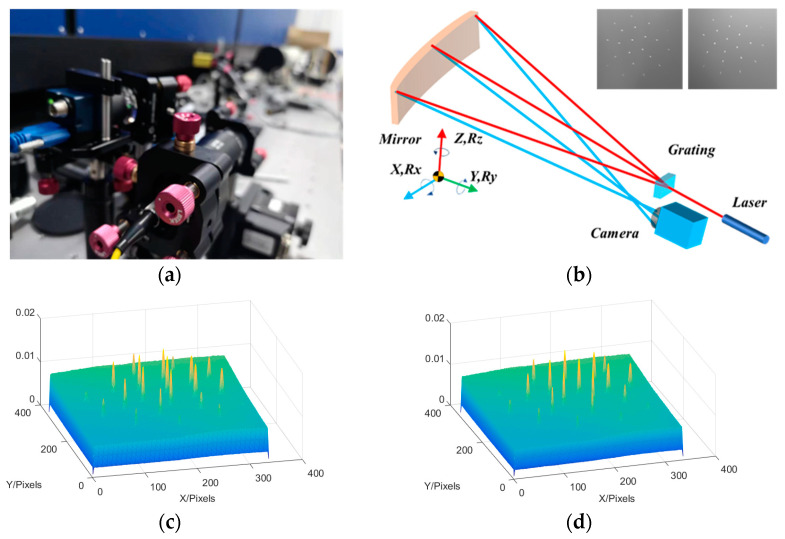
System optical axis data based on the optical truss: (**a**) Experimental site and (**b**) Schematic of the experimental optical path and target image. (**c**) Position distribution of light points before grating rotation. (**d**) Distribution of light point positions after grating rotation.

Using optical trusses, the optical axis alignment accuracy could be maintained at 2 mrad, and the change in the system pointing angle during pentaprism scanning had to be less than this value. For high-precision measurements, rotation measurements could reduce the shaking caused by torque imbalances and achieve high detection accuracies. The scene diagram of the system’s optical axis alignment and the correspondence principle diagram are shown in Figure 5a. The optical reference of the system can be established using this light intensity distribution. Specifically, the exit point of the grating is set as the center of curvature of the system. By rotating the grating, the corresponding light with the smallest change in the reflected position of the light point can be considered as the optical axis of the system, and based on this, a benchmark for maintaining system stability is established.

### 4.2. System Wavefront Measurement

**Figure 6:**
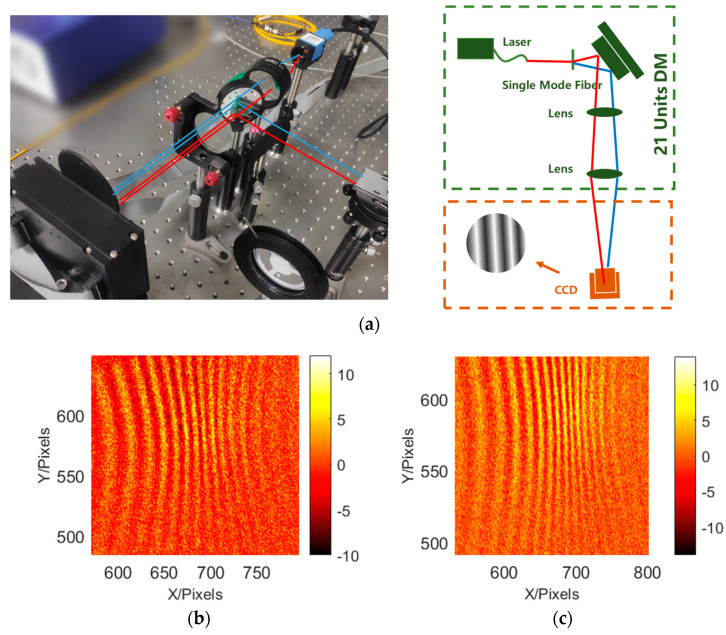
Verification of wavefront testing: (**a**) system set-up and (**b**,**c**) fringe intensities before and after modification, respectively.

**Figure 7:**
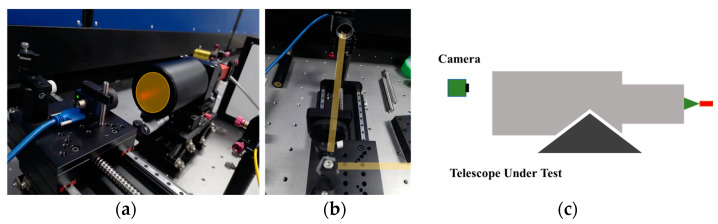
Verification platform for the interferometric projection system: (**a**) direct light intensity detection, (**b**) detection using a pentaprism, and (**c**) system schematic.

**Figure 8:**
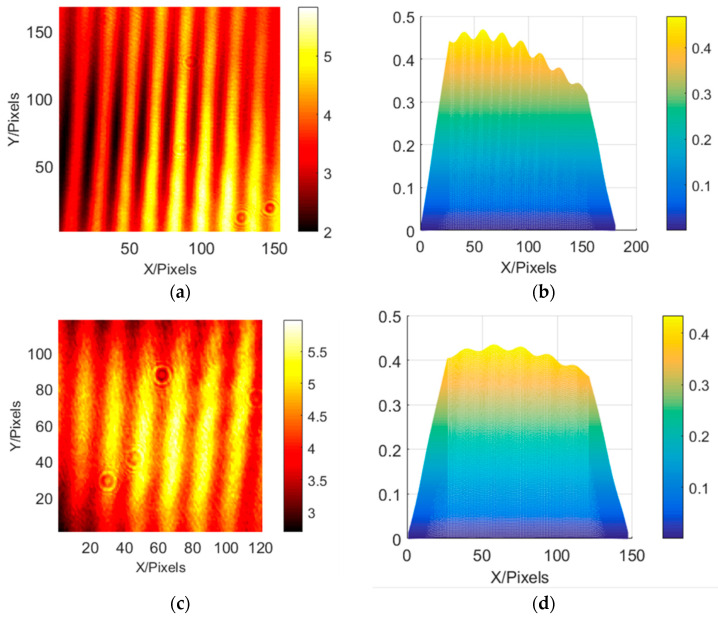
Comparison of light intensities between direct detection and scanning detection: (**a**,**b**) direct detection interference fringes and fringe positions obtained by correlation operation, respectively; (**c**,**d**) the interference fringes collected by scanning and fringe positions obtained by correlation operation, respectively.

In this paper, the method of direct camera detection and pentaprism pupil fringe detection is used to verify the system’s fringe acquisition. The results of the system’s direct acquisition and pentaprism scanning acquisition are similar, so the pentaprism polarizer or the camera can be used to collect stripes according to the actual situation. Based on the principle of shear interference [23], the spacing of system fringes is related to local tilt. Therefore, the local slope of the system can be obtained by changing the fringe spacing of each sub aperture, and the system’s wavefront can be reconstructed based on the slope Astigmatism was introduced at one wavelength using a deformable mirror. Results of change in the fringes is shown in Figure 6. It can be concluded that the fringes are staggered by 0.9 wavelengths, indicating that the wavefront sensing accuracy is better than 1/10 of the working wavelength (633 nm). The verification platform for the interferometric projection system is shown in Figure 6. The comparison of light intensities between direct detection and prism-free detection is shown in Figure 7 and Figure 8.

### 4.3. System Spectral Response Measurement

**Figure 9:**
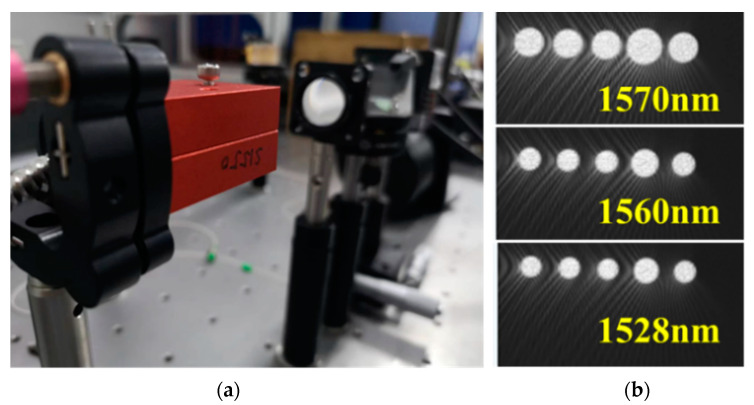
Verification of high-flux system spectral testing: (**a**) system installation and (**b**) spatial distribution light intensities at different wavelengths.

The spectral reception capability of the system was verified using a sweep frequency laser to match the sub-spectral bands and multi-mode fiber confocal system for light acquisition. High-flux spectral detection was achieved using integrated photonic devices. Five optical waveguides were integrated to obtain the spectral responses under different spectral inputs. The system used a broadband light source to output broadband light. The output band was selected using a wavelength division multiplexer and the light of the selected band was sent to the system’s focal plane using a coupler. During operation, the center position of the projection was verified using the waveguide of the center position and lens set; moreover, the system projection range was verified using the edge waveguide. After calibrating the transmittance of each waveguide based on the central wavelength (1560 m), the uniformity of the system’s light intensity was better than 5%. We made sure the coupling efficiency was high at each position on the pupil surface, ensuring that the laser power used did not induce nonlinear effects. The verification of the high-flux system spectral testing is shown in Figure 9.

## 5. Discussion

Considering the increasing complexity of telescopic systems, the mechanical and optical reference transfer between multiple subsystems influences the registration accuracy and fusion effects of multi-modal joint measurements. Therefore, traditional methods are no longer suitable for the current calibration requirements of optical systems with large apertures offering a “large span, multiple parameters, and a common reference,” and research into a new system for the in-situ measurement and detection of large-aperture telescopes is urgently needed.

The optical axis could be precisely measured using PSP. Using the high-quality collimated beam incident on the pupil surface, we performed calibrations by combining the ideal target center positions of different terminals. Meanwhile, when components such as the secondary mirrors were tilted, the position of the light spot formed by the system changed. The tilt of the system’s optical axis could effectively be identified based on these changes.

Using optical axis and spectral response measurements, the optical lever effect (for short focus systems, the focal length can be extended via the 4f system) can be applied to measure the slope of the wavefront through the focal plane to obtain the optical surface quality of the system and relative position of the optical components. The polar and Cartesian coordinate system scanning modes can be selected when applying the actual system. Aiming for a highly efficient test process, rotation sampling is applied to maximize the accuracy and minimize the time; the overall planning of the sampling locations, scanning paths, and dwell times are optimally designed by balancing the spatial resolution, detection efficiency, and detection accuracy.

PSP could be used to perform local sampling of the pupil based on the mapping relationship between the focal and pupil planes. Flexible sampling and reconstruction of the system parameters could also be achieved. We could transform phase measurements into energy measurements using spatial light intensity projection and the photon pentaprism. We could also construct an efficient in-situ detection system offering high flexibility, a small size, and minimal external influences on the baseline configuration of the photon pentaprism.

## 6. Conclusions

Telescopes with high spatial resolutions and detection capability are expected to offer high sensitivities; high spatial, temporal, and spectral resolutions; and large fields of view (FoV). In response to the calibration requirements for in-situ detection of multiple parameters (wavefront, optical axis, transmittance, etc.) in large aperture optics systems, utilizing the characteristics of fiber-interconnections such as small volume, light weight, and high degree of freedom in light transmission, high-precision in-situ calibration of multiple parameters is ultimately achieved. The measurement accuracy of the optical axis of the system is better than 2 mrad. The spectral measurement accuracy is better than 5%, and the wavefront measurement accuracy surpassed 0.1 wavelengths (1 wavelength = 633 nm). The Photonics Scanning Pentaprism (PSP) System is investigated here, and it can ensure the performance of the system under in-site environment and at long-term.

## Data Availability

The data underlying the results presented in this paper are not publicly available at this time, but may be obtained from the authors upon reasonable request.
